# Relevance of Regulatory T Cells during Colorectal Cancer Development

**DOI:** 10.3390/cancers12071888

**Published:** 2020-07-14

**Authors:** Jonadab E. Olguín, Itzel Medina-Andrade, Tonathiu Rodríguez, Miriam Rodríguez-Sosa, Luis I. Terrazas

**Affiliations:** 1 Laboratorio Nacional en Salud, Diagnóstico Molecular y Efecto Ambiental en Enfermedades Crónico-Degenerativas, Facultad de Estudios Superiores (FES) Iztacala, Universidad Nacional Autónoma de México (UNAM), Av. De los Barrios # 1, Tlalnepantla 54090, Mexico; efra004@gmail.com (J.E.O.); itzelmedina.andrade@gmail.com (I.M.-A.); tonathiurh@hotmail.com (T.R.); rodriguezm@unam.mx (M.R.-S.); 2 Unidad de Biomedicina, FES Iztacala, UNAM, Av. De los Barrios # 1, Tlalnepantla 54090, Mexico

**Keywords:** regulatory T cells, colorectal cancer, animal models, clinical trial, phenotypic plasticity

## Abstract

In recent years, there has been a significant increase in the study of own and foreign human factors favoring the development of different types of cancer, including genetic and environmental ones. However, the fact that the immune response plays a fundamental role in the development of immunity and susceptibility to colorectal cancer (CRC) is much stronger. Among the many cell populations of the immune system that participate in restricting or favoring CRC development, regulatory T cells (Treg) play a major role in orchestrating immunomodulation during CRC. In this review, we established concrete evidence supporting the fact that Treg cells have an important role in the promotion of tumor development during CRC, mediating an increasing suppressive capacity which controls the effector immune response, and generating protection for tumors. Furthermore, Treg cells go through a process called “phenotypic plasticity”, where they co-express transcription factors that promote an inflammatory profile. We reunited evidence that describes the interaction between the different effector populations of the immune response and its modulation by Treg cells adapted to the tumor microenvironment, including the mechanisms used by Treg cells to suppress the protective immune response, as well as the different subpopulations of Treg cells participating in tumor progression, generating susceptibility during CRC development. Finally, we discussed whether Treg cells might or might not be a therapeutic target for an effective reduction in the morbidity and mortality caused by CRC.

## 1. Introduction

Traditional treatments to reduce the mortality of cancer, such as surgery, radiotherapy, or chemotherapy, have shown limitations in their effectiveness. In recent years, emphasis has been placed on the importance of the immune response as an orchestrator of resistance for different types of cancers [1]. Immunotherapy is a way of reducing the mortality caused by cancer [2], for example, the adoptive transfer of antigen-specific T cells, which has been shown to generate a positive immune response in metastatic melanoma [3]. The use of monoclonal antibodies directed against specific molecular and cellular targets, as well as the use of immunotherapy based on T cell clones in gastric cancer, are now relatively affordable [2,4]. It has been shown that the use of NK cells activated with IL-2 during acute myeloid leukemia can generate complete remission in 26% of patients [5]. The use of anti-PD-1 and programmed cell death ligand 1 (PD-L1) antibodies have shown long-lasting responses in non-small cell lung cancer, with a favorable safety profile and manageable side effects [6]. During colorectal cancer (CRC), some immunotherapy clinical trials have demonstrated a potential benefit, but most of them remain as experimental options. Vaccines directed against overexpressed peptides in mucosal tissues of patients with CRC, which can induce tumor antigen-specific immune response with a lower risk of inducing autoimmunity, have been used [7]. Dendritic cell transfer pulsed with tumor epitopes or tumor cell lysates have been used to induce immunity to gastric cancer [7]. As mentioned above, some immunologic strategies have been used as treatment against some types of cancers, showing effectiveness, but continuous research development is necessary for a clear panoramic understanding of the role of the immune response during CRC.

## 2. Regulatory T Cells: Brief Summary

Since regulatory T (Treg) cells were described in 1995 by Shimon Sakaguchi, they have been protagonists in most immunological processes caused by self or non-self antigens in parasitic, autoimmune, inflammatory, and oncological diseases [8]. First, Treg cells were described as a subpopulation of T cells that suppressed the immune response, avoiding autoimmunity, and were characterized by the expression of the alpha chain receptor for IL-2 [9], also called CD25, which had previously been found expressed on activated T cells [10,11]. After a long race to determine if a specific marker for Treg cells exists, it was shown that the forkhead box p3 transcription factor (Foxp3) is the molecule conferring suppressor activity on Treg cells [12,13]; thus, Foxp3 is the master regulator of Treg cells [14]. Mice and humans with a mutation in the *Foxp3* gene display a T cell-dependent, lymphoproliferative immune disorder manifested by some diseases, such as type-1 diabetes, thyroiditis, splenomegaly, and lymphadenopathy [15].

**Figure 1:**
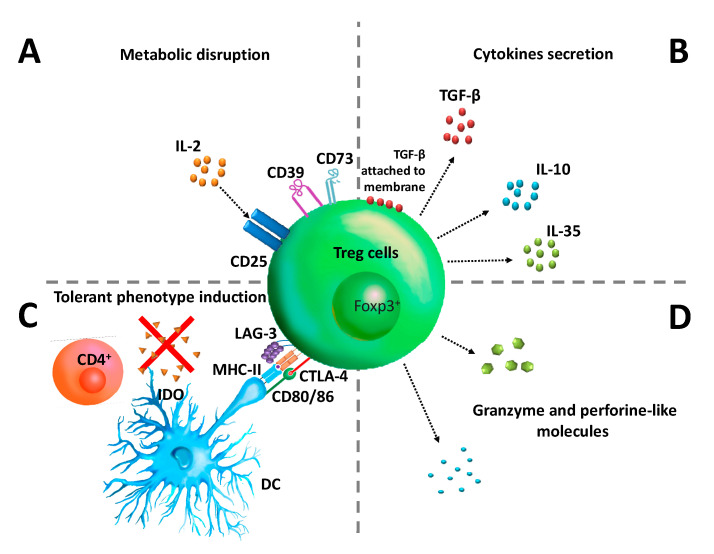
Natural regulatory T (Treg) cells and their main suppressive mechanisms. (**A**) Metabolic disruption of IL-2 caused by an increased expression of CD25 (high-affinity IL-2 receptor) in Treg cells, also caused by the release of extracellular adenosine. (**B**) Secretion of cytokines such as IL-10, TGF-β, and IL-35. (**C**) Manipulation of antigens presenting cells for a tolerant phenotype. (**D**) Secretion of granzyme and perforin.

Treg cells use several mechanisms to suppress immune responses, such as deprivation of IL-2 by its IL-2 (CD25) high-affinity receptor (Figure 1A) [16,17,18,19], the use of CD39 and CD73 ectoenzymes for the release of extracellular adenosine (Figure 1A), which is a strong immunosuppressant [20,21,22], the secretion of suppressor cytokines such as IL-10 [23], TGF-β [24,25] and IL-35 [26,27] (Figure 1B), the manipulation of antigen-presenting cells by inducing a “tolerant phenotype” through Cytotoxic T-Lymphocyte Antigen 4 (CTLA-4), and the Lymphocyte Activation Gene-3 (LAG-3) to induce the Indoleamine 2,3-dioxygenase (IDO) enzyme, which in turn reduces the availability of tryptophan in the environment along the kynurenine pathway (Figure 1C) [28,29,30]. In humans, it has also been reported that Treg cells use granzyme and perforin-like molecules as a suppressive mechanism (Figure 1D) [31,32].

**Figure 2:**
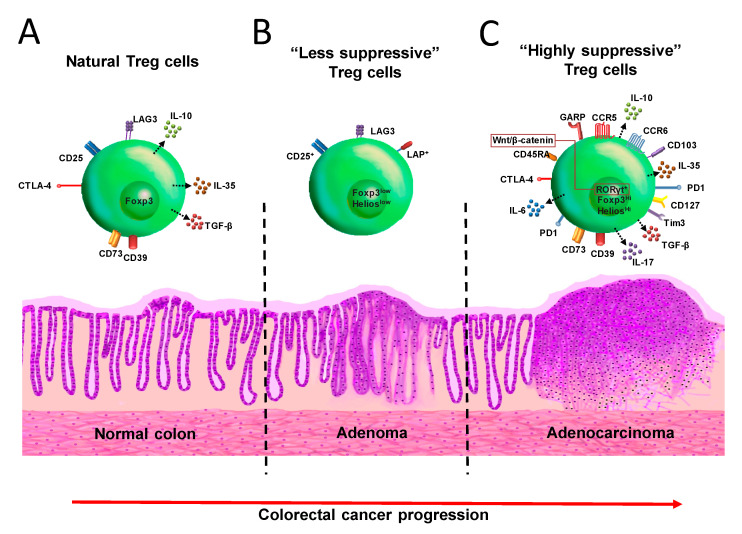
Phenotype of Treg cells in the progression of CRC. As mentioned in the text, adenomas are the precursors of CRC, arising from the adenoma-carcinoma sequence. (**A**) When the intestinal tissue has a normal condition, natural Treg cells display a regular phenotype, but the genetic, epigenetic, and mainly the immunological alterations that end in the formation of adenomas, modify the phenotype in Treg cells, which confers different roles, depending of the grade of alterations during CRC. We included these subpopulations of Treg cells in 2 groups: (**B**) “less suppressive” Treg cells which are associated with an immunological protection against tumor formation, and (**C**) “Highly suppressive” Treg cells, whose phenotype is associated with tumor progression and a poor protective immune response against CRC.

Besides the expression of CD25 and the Foxp3 transcription factor, Treg cells also display some molecules associated with activation in their surface, which confer on them a higher suppressive capacity, such as Glucocorticoid-Induced Tumor Necrosis Factor receptor (GITR), Cytotoxic T-Lymphocyte Antigen 4 (CTLA-4), Inducible T-cell Costimulator (ICOS) [33], Programmed cell Death protein 1 (PD-1) [34], and T-cell immunoglobulin and mucin-domain containing-3 (Tim-3) [35] (Figure 2A). All these features make Treg cells a versatile immune population with a wide range of mechanisms that could be manipulated either for or against the protection of health.

## 3. Treg Cells during CRC in Clinical Cases: An Overview

Colorectal cancer is one of the most common and fatal cancers in the world [36], being the third most common cancer worldwide, and the second most deadly, just behind lung cancer [37]. The incidence rates are higher in developed countries, but the mortality rate is much higher in developing ones [38,39,40]. CRC has different origins: hereditary, sporadic, and colitis-associated colon cancer (CAC). Only 5% of CRC cases are hereditary, whereas 75% are sporadic, being associated with environmental factors, and the remaining 20% are associated with dysregulated inflammatory responses in the colon [41]. An aggressive and prolonged inflammation generates adenomas in colon tissues [42]. Colorectal adenomas are lesions with dysplastic epithelium, characterized by being benign in general, and dysplasia that could be either low or high is mainly determined by abnormal nuclear morphology [43]. However, adenomas are the precursors of CRC, arising from the adenoma-carcinoma sequence; the risk of malignancy increases along with polyp size and degree of dysplasia [43]. Both genetic and epigenetic alterations contribute to the formation of immunogenic tumor-specific and tumor-associated antigens [44], which allow the identification and elimination of CRC by the immune response [45,46]. However, some cells of the immune response play a role in the initial inflammation, resulting from tumor initiation up to metastasis during CRC. For example, myeloid cells, such as monocytes, macrophages, and neutrophils, secrete cytokines and express immunomodulatory molecules in their surface, which may promote the development of the tumor and avoid its elimination, through the induction and maintenance of an immunosuppressive microenvironment [47,48].

Similarly, aiming to induce immunosuppression, Treg cells have been associated with tumor progression during CRC (Figure 2) [49]. Interestingly, CRC patients display Treg cells with a higher expression of several molecules that correlate with suppression, such as Tim-3, LAG-3, TGF-β, IL-10, CD25, and CTLA-4 [50], and by the BLIMP-1 transcription factor expression in the tumor [51]. The infiltration of Treg cells into the colon is significantly higher in CRC than in a healthy colon, as well as in patients with limited disease (Union for International Cancer Control (UICC) criteria I and II) than in metastatic (UICC criteria III and IV) ones [52]. This was also associated with the frequency of Foxp3^+^ cells in patients with lymphatic invasion [53]. The increase in Foxp3 expression in colorectal tissues and peripheral blood correlates with an increasing degree of tumor malignancy and lymph-node metastasis [54]. It was also demonstrated that patients with CRC had increasing percentages of Treg cells in the peripheral blood and mesenteric lymph nodes compared to either healthy controls or patients with inflammatory bowel disease. Additionally, in this study, it was observed that when Treg cells from CRC patients were depleted in peripheral blood, CD4^+^ cells produced IFN-γ in a specific-antigen shape against the tumor antigen 5T4 but not in the control samples [55]. Therefore, Treg cells can inhibit an anti-tumor specific immune response in patients with CRC. Another study showed that the density of Treg cells was dramatically higher in tumor-draining lymph nodes than in peripheral blood or tumor-infiltrating lymphocytes, and these data were correlated with the staging of the disease. Furthermore, the CD8^+^ T cell function was restored after Treg depletion [56]. More recently, it was found that patients with colon adenocarcinoma displayed an accumulation of Treg cells overexpressing PD-1, which impaired CD8^+^ T cells activity in situ [57]. Treg cells in tumor patients are specific for a limited repertoire of tumor antigens, suggesting that these cells exert strong T cell suppression in an antigen-selective manner during CRC, and the effector/memory T cell response against antigens recognized by Treg cells strongly increases after Treg cell depletion [58]. Thus, the evidence for a role of Treg cells as part of the immunosuppressive microenvironment that promotes the development of tumors during CRC has been described, and it suggests that the frequency of sub-populations of Treg cells may provide a useful tool with possible prognostic value for the treatment of CRC.

## 4. Treg Cells during CRC in Murine Models

The use of animal models is a tool that grants easier access to samples, for the quantity of samples and for the short period of time it takes to obtain results. Many ideas applied in the clinic came from experiments designed and performed in murine models. With this in mind, the study of Treg cells has been applied to CRC in murine models. One model of CRC in mice was described by Tanaka in 2003, where azoxymethane (AOM) and dextran-sodium sulfate (DSS) were used. Mice were injected with AOM (which exerts colonotropic carcinogenicity), 12.5 mg/kg; then, 7, 29, and 51 days after AOM injection, 2% DSS (for colitis induction) was added to the drinking water for 7 days. This model is called CAC and it displays many similarities to CRC in humans, but with the advantage of obtaining results faster. It has been demonstrated that at the final stage of CAC development [59], Treg cells increase in number and exhibit a phenotype of activation defined by the expression of CD103, Receptor Glycoprotein-A Repetitions Predominant (GARP), CTLA-4, and IL-10 (Figure 2C) [60]. This is in accordance with the idea that a correlation between Foxp3 expression and tumor progression during CRC exists [61]. The transient ablation of Treg cells using depletion of Treg cells (DEREG) mice, which express the diphtheria toxin receptor under the Foxp3 promotor [62], suppressed colon tumor size; however, the mortality rate for these mice increased [60]. In our lab, we were interested in the dynamic behavior of Treg cells during the development of CAC, focusing not only on the final phase of tumor formation but also on all the phases of its development. Using transgenic Foxp3^EGFP^ mice and the CAC model, we observed a reduced percentage of Treg cells in blood and spleen during early CAC development, and an increased percentage of these cells was shown at late stages of CAC in mesenteric lymph nodes (MLN) [63]; these observations have been confirmed in patients with CRC [64]. Conversely, at early stages of CAC, a higher percentage of activated T cells (Tact) were observed, but as CAC progressed it was detected that Tact cells were significantly reduced. Interestingly, Treg cells from late stages of CAC displayed an activated phenotype featured by increased expression of PD-1, Tim-3, and CD127 molecules in their membranes (Figure 2C). Moreover, these Treg cells from CAC mice obtained from MLN suppressed CD4^+^ and CD8^+^ T-activated cells in a more efficient way than healthy wild-type mice. Thus, with the idea of inhibiting the accumulation of Treg cells during CAC development, we used the PC61 monoclonal antibody (anti-CD25) during the early phase of CAC development to reduce the percentage of Treg cells; this early intervention guarantees the reduction mainly in Treg and not in Tact cell population [63]. Reduction of 50% of Treg cells resulted in a better prognostic value by a significant reduction in the tumor load, which was associated with an increased percentage of both CD4^+^ and CD8^+^ T-activated cells in MLN in CAC mice receiving immunotherapy with the monoclonal antibody PC61. All these results suggest that Treg cells play a critical role by suppressing the immune response in the early stages of CAC development [63].

The inoculation of colorectal carcinoma tumor cells has also been used for the study of Treg cells during CRC development in mouse models. In this orthotopic mouse model, it has been reported that Treg cell depletion using PC61 antibody before the inoculation of colorectal carcinoma tumor cells CT26 resulted in protective immunity mediated by CD8^+^ cytotoxic T cells; thus, the specificity of cytotoxic responses to tumor antigens can be suppressed by Treg cells [65]. As mentioned above, Treg cells express the ecto-enzyme CD73, which together with CD39 can hydrolyze the extracellular ATP in adenosine, a strong immunosuppressant. It is known that extracellular adenosine is accumulated in the tumor microenvironment, suppressing the anti-tumor immune response (Figure 2C). Adenosine levels can increase in response to chronic inflammation, which is a characteristic of CRC. Thus, CD73 ablation significantly suppressed the growth of the MC38 colon cancer cell line, in a CD8^+^ T cell-dependent pathway. This effect was associated with an increased level of both antigen-specific CD8^+^ T cells and IFN-γ production in peripheral blood and locally in the tumors [66].

In the *adenomatous polyposis coli* (APC) animal model of human familial adenomatous polyposis (FAP), mice develop numerous polyps in the intestinal tract due to a truncation in the APC gene [67,68]. This model has been used as a tool for the evaluation of anticancer, chemo-preventive agents, and also for the study of the immune response against CRC [67,68]. During the CRC development in the APC^min/+^ mouse model, CD4^+^Foxp3^+^ Treg cells accumulate in the adenomas, which match with lower frequencies of conventional T and B cells in situ [69], indicating a downmodulation of the local immune response against CRC. Furthermore, in this model, adenomas displayed an altered chemokine profile with high levels of CCL17 and low levels of CXCL11 and CCL25, and their Treg cells did not express CXCR3 [69]. By breeding APC^min/+^ mice with DEREG mice (see above), it was possible to selectively deplete Treg cells in tumor-bearing mice. This exclusive depletion of Treg cells increased the frequency, infiltration, and proliferation of T cells in the tumors, which correlated with increased expression of CXCR3^+^ T cells and IFN-γ production [70,71]. CXCR3 and its ligands are differentially expressed at sites of inflammation and within the CRC tumors; CXCR3 is functionally expressed on Treg cells and also induces the differentiation of peripheral T cells into Treg cells, suggesting that a CXCR3 molecule could be an indirect Treg cell target with therapeutic potential during CRC [72]. In an APC^min/+^ murine model, it was demonstrated that the oral administration of IL-10 encapsulated in microparticles reduces polyposis and increases the survival rate, apparently this controversial IL-10 effect can be explained given that IL-10 has a neutralization effect over Foxp3^+^RORγt^+^IL-17^+^ Treg cells that promote the disease and a positive effect on the restauration of Foxp3^+^RORγt^−^IL-17^−^ Treg cells, which are protective (Figure 2C) [73]. Recently, in the APC^min/+^/DEREG mouse model, it was shown that Treg cells specifically suppressed the TCRαβ^+^ CD8^+^ T cell population in colon tumors; when Treg cells were depleted, an increased amount of granzyme B and IFN-γ was observed in CD8^+^ T cells [74]. In another study, it was demonstrated that the adoptive transfer of T cells secreting IL-10 attenuated microbial-induced inflammation, suppressing polyposis in APC^∆458^ mice. In contrast, the ablation of IL-10 specifically in T cells produced pathologies like in systemic IL-10 deficient mice, increasing the number and growth of colon polyps. Treg cells and T cells are the major source of IL-10 in healthy colons and in colons containing polyps in this model [75]. Additionally, mice receiving broad-spectrum antibiotics presented a reduction in the microbiota, inflammation, and polyposis, suggesting that polyposis is fueled by a high number of microbes that accumulate in the colon, which in turn activate the inflammatory response; this inflammation is suppressed by IL-10 secreted by T and Treg cells [75]. It is clear that Treg cells play a role in the promotion of tumor development and in the reduction in an efficient immune response, but the results described above, where IL-10 from Treg cells play a role in the reduction in colon damage [73,75], are remarkably contrasting. These controversial findings could probably be explained by the fact that many subpopulations of Treg cells secreting different kinds of cytokines have different roles during CRC in both clinical and mouse models. However, these models highlight the importance for the microbiota in the context of CRC and Treg cells, because the higher densities of microbes that accumulate within polyps trigger local inflammatory responses, which are suppressed by IL-10 derived from both T and Treg cells. All these results suggest a close relationship between Treg cells and microbiota promoting tumor development during CRC.

## 5. Subpopulations of Treg Cells during CRC

In the past, evidence was collected suggesting that once a CD4^+^ T-lymphocyte acquired T_H_1 (mainly secreting IFN-γ and inflammatory cytokines) or T_H_2 (mainly secreting IL-4, and inhibitory T_H_1 cytokines, promoting the antibody secretion) phenotypes, it was permanent during the functional life-span of the CD4^+^ T cell [76]. However, some years ago evidence emerged supporting the idea that CD4^+^ T cells actually can change their phenotype and function, being more flexible than expected in the production of cytokines [77]. This is because transcription factors such as T-bet, GATA-3, RORγt, and Foxp3 are expressed transiently, or because the CD4^+^ T-cells can express more than one transcription factor at the same time [78]. This kind of phenotypic plasticity was not only described in CD4^+^ T cells but also proposed in myeloid cells, including macrophages, mast cells, and neutrophils [79]. Treg cell phenotypic plasticity has also been suggested during CRC development, where an increased number of Treg cells Foxp3^+^ expressing IL-17 during human CRC was shown. These Treg cells also expressed CCR6^+^ TGF-β^+^ and IL-6^+^ (Figure 2C). This population, called Foxp3^+^IL-17^+^ Treg cells, was more suppressive against CD8^+^ T-activated cells, and surprisingly, this suppression was reversed in the presence of an anti-IL-17 blocking antibody [80]. Foxp3^+^IL-17^+^ Treg cells are selectively accumulated in the colonic microenvironment associated with colon carcinoma, and these types of Treg cells also favor inflammatory cytokine production in colon tissues. These data suggest that Foxp3^+^IL-17^+^ Treg cells probably facilitate a chronic inflammatory pathological microenvironment in the colon, thus promoting tumor development [81]. In line with this evidence, a preferential expansion of a Foxp3^+^RORγt^+^ subpopulation of Treg cells emerges in human CRC. These cells show a potent suppressive capacity but with an anti-inflammatory compromised ability (Figure 2C). These Foxp3^+^RORγt^+^ Treg cell populations with the same suppressor abilities were shown in both a mouse model of hereditary polyposis [82] and an AOM/DSS CAC model [83]. The specific ablation of RORγt gene in Foxp3^+^ T cells improved the polyp-specific immune surveillance and attenuated the polyposis, indicating the inflammatory activity of these cells. Interestingly, the ablation of IL-6, IL-23, or IL-17 reduced the number of polyps but not in the same way that ablation of the RORγt gene did [84]. It was demonstrated that Wnt/β-catenin signaling in T cells promotes the expression of RORγt, which in turn promotes Th17-mediated inflammation (Figure 2C). In addition, the expression of β-catenin is increased in Treg cells from both mice and patients with CRC. The activation of β-catenin only in Treg cells was enough to generate inflammation and carcinogenesis [85]. This evidence supports the idea that a subpopulation of Treg cells with a T_H_17-like profile may exert both powerful inflammatory damage and strong immunosuppression during CRC development.

We previously mentioned that some studies on Treg cells are controversial, because the presence of Treg cells and their suppression mechanisms are involved in either a better or worse prognosis during CRC [73,75,86]. Due to this evidence, we think that some subpopulations of Treg cells likely have different roles at the same time. In fact, it has been demonstrated that during CRC development, Treg cells could be classified into two subpopulations by the grade of Foxp3 expression in Foxp3^lo^ or Foxp3^hi^. The Foxp3^lo^ Treg cells are not suppressive (Figure 2B), and do not express the CD45RA receptor but secrete inflammatory cytokines. CRC patients with abundant Foxp3^lo^ Treg cells showed a significantly better prognosis [87]. In another study, it was demonstrated that most of the intra-tumor CD4^+^Foxp3^+^ Treg cells have a Helios^+^, CTLA-4^+^, and CD39^+^ phenotype, but 30% of CD4^+^Foxp3^−^ cells also expressed markers associated with regulatory functions, including CD25, LAG-3, and latency-associated peptide (LAP) (Figure 2B). This adaptive Treg subpopulation also produced IL-10 and TGF-β, and it was 50 times more suppressive than CD4^+^Foxp3^+^ Treg cells [88]. Helios is a member of the Ikaros transcription factor family and is preferentially expressed in Treg cells. It has been suggested that Helios is a marker for thymus natural Treg cells and not for induced Treg cells [89]. Helios^low^Foxp3^+^ Treg cells are enriched both in peripheral blood and at the tumor site (Figure 2B), but only Helios^hi^Foxp3^+^ Treg cells accumulate significantly and specifically in tumors (Figure 2C) and produce IL-17 during CRC. CD4^+^Foxp3^+^Helios^+^ Treg cells co-expressed the suppressive molecules PD-1, CTLA-4, and CD39, suggesting that the phenotype of these Treg cells in the tumor of CRC patients is highly suppressive (Figure 2C) [90]. The manipulation of subpopulations of Treg cells, such as Foxp3^lo^/Foxp3^hi^ Treg cells co-expressing Helios, LAP, or another molecule associated with immunosuppression, could be useful targets to develop a strategy for an effective fight against CRC.

Another molecule directly associated with the suppressive activity of Treg cells is the IL-35 cytokine, which confers regulatory activity on naïve CD4^+^ T cells and also suppresses T-cell proliferation [26,27]. Interestingly, IL-35 levels have been found to be elevated in both serum and tumors in patients with CRC, and they were correlated with tumor metastasis. Moreover, Treg cells from CRC patients were also capable of secreting high levels of IL-35 (Figure 2C) [91].

The chemokine receptor CCR5 has been involved in the recruitment of systemic Treg cells during CRC, and CCR5^−/−^ mice have a delayed tumor growth because a reduced number of Treg cells are infiltrated in the tumors [92]. In human CRC, functional CCR5 was highly expressed in tumor-infiltrating Treg cells, and Treg cells expressing high levels of CCR5 are more suppressive; however, the pharmacological inhibition of CCR5 failed to reduce the tumor-infiltrating Treg cells, suggesting that other chemokine receptors are probably involved in the recruitment of Treg cells into the tumor during CRC development [93]. Perhaps a combined immunotherapy seeking to block two or more targets may favor a better prognosis against CRC in the clinic, but the establishment of more feasible therapeutic targets to improve the immunomodulation in CRC is clearly necessary.

## 6. Immune Molecules and Cells Promoting or Inhibiting Treg Cell Activity during CRC

We have described above the different suppression modes of Treg cells during CRC and how Treg cells are classified into subpopulations with specialized immunosuppressant features (Figure 2). Next, we are going to establish that Treg cells and several molecules and immune cells are involved in the promotion or inhibition of Treg cells during CRC in animal models and in clinical trials.

### 6.1. TGF-β1, Runt-Related Transcription Factor (RUNX) 3, and GARP

**Figure 3:**
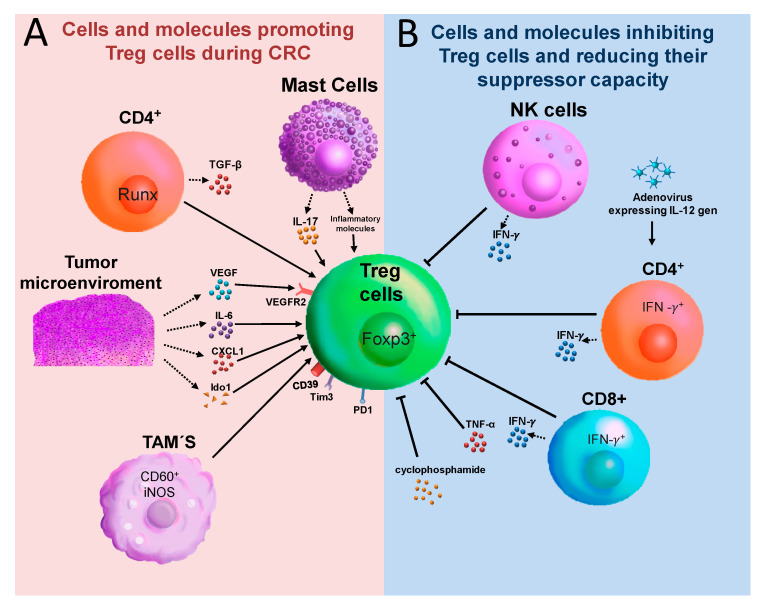
Different molecules and cells either promoting or inhibiting Treg cell activities during CRC. (**A**) As described in the text, some cells, such as mast cells, T cells, and TAMs, are involved in the induction of Treg cell population during CRC development. Additionally, the same tumor microenvironment produces molecules to induce a more suppressive population of Treg cells. (**B**) On the other hand, cells such as NK, CD4, and CD8 T cells, are involved in the inhibition of the suppressor capacity of Treg cells during CRC.

TGF-β1 is essential for the maintenance of inflammatory homeostasis; loss of its signaling is involved in malignant tumor formation [94]. RUNX is a family of proteins that participate down-stream of TGF-β1 signaling, and their loss is involved in severe inflammation and tumor formation in the gastrointestinal tract [95]; RUNX3 is involved in the differentiation of CD8 and NK cells; thus, Runx3^−/−^ mice develop immunodeficiency by the absence of these cells [96]. Using stylish experiments with chimeric mice, it has been demonstrated that the loss of RUNX3, specifically in T cells, resulted in an impaired suppressive ability of the Tregs, as well as a reduction in the numbers of induced Treg cells by TGF-β stimulation (Figure 3A). This lack of activity of Treg cells caused colitis and the development of tumors in the large intestine and cecum, when animals were housed in a conventional animal facility. In the same way, no tumor was detected when CD8^+^ T or Treg cells from WT origin were transferred into chimeric mice; however, the tumor formation was completely blocked by housing animals in a pathogen-free condition, suggesting that microbiota is involved in tumor development [97]. These results are controversial with data where Treg cells are involved in the promotion of tumor growth and reduced immune response, which leads to the development of CRC. Probably these data represent an example of a subpopulation of Treg cells acting against tumor formation. The GARP molecule expressed on the surface of Treg cells is involved in the activation of TGF-β signaling; thus, the specific absence of GARP in Treg cells in a CAC model improved anti-tumor immunity [98].

### 6.2. IL-33 Receptor

Another interesting molecule is the IL-33 receptor (ST2 or IL1RL1), which is involved in the stimulation of the suppressive functions of Treg cells in physiological and pathological conditions [99]. ST2 is highly expressed in tumors of CRC patients, which correlates with an increased expression of Foxp3 in both adenoma and CRC tissues. A higher density of ST2 expression in tumor samples is associated with increased dysplasia [100].

### 6.3. Vascular Endothelial Growth Factor (VEGF)

A molecule involved in Treg cell promotion is VEGF, which is both secreted and abundant in the tumor microenvironment and suppresses anti-tumor immunity. Its receptor, VEGFR2, is expressed selectively in intra-tumor Treg cells with high expression of Foxp3 (Figure 3A) [101], and it is proposed that both VEGFR2 and Foxp3 may be better predictive markers for recurrence and survival in patients with CRC [101].

### 6.4. Indoleamine 2,3-Dioxygenase (IDO)

In different types of tumors, the IDO enzyme expression has been found to be increased in tumor tissue and in draining lymph nodes, and it is believed that it plays a role in tumor evasion by suppressing the immune response [28]. Ido1^−/−^ mice (Ido1 is a paralog enzyme involved in the degradation of tryptophan) either bred with APC^min/+^ mice or in the CAC model did not lead to significant differences in the size and number of colon tumors. However, Ido1 deficiency altered the immune response in the tumor microenvironment with increased levels of pro-inflammatory cytokines and a reduced number of Treg cells (Figure 3A). Thus, the exclusive elimination of IDO is not sufficient to reduce the progression of colon cancer [102].

### 6.5. CD39

As previously mentioned, CD39 is an ecto-enzyme that mediates the generation of immunosuppressive adenosine [20,21]. Treg cells from CRC patients express high levels of CD39 [103], which may increase the amount of adenosine available in the tumor microenvironment. Adenosine reduces the capacity of monocytes to activate the endothelium, which indirectly affects T cells’ recruitment to reach the site of the tumor. Thus, Treg-derived adenosine acts on monocytes and not only helps reduce trans-endothelial activation, but also affects the migration of effector T cells during CRC (Figure 3A) [104]. Moreover, the gene expression profiles in CD4^+^CD25^+^Foxp3^+^CD127^low^ Treg cells was described in patients with CRC. The genetic profiling analysis led to the identification of 61 immune-related genes in Treg cells. Most of these genes were involved in cytokine/chemokine mediators of inflammation, chemokine receptors, lymphocyte activation, and TCR receptor signaling pathways, CCR1, CCR2, IL-10, and SOCS3 [105] being the most relevant. Thus, molecules such as IDO, VEGFR, Runx3, and TGF-β contribute to the promotion of Treg cells during CRC.

### 6.6. Tumor-Associated Macrophages (TAMs)

TAMs are an important cell component in the microenvironment of various types of solid tumors; in CRC, TAMs are involved in tumor initiation and metastasis [47,48]. Moreover, CD60^+^INOS^−^ TAMs that infiltrated tumors of CRC patients have been associated with increased CD8^+^Foxp3^+^ Treg cells in the tumor stroma, being a negative prognostic factor in patients with CRC (Figure 3A) [106]. Moreover, TAMs can recruit CCR6^+^Treg cells through CCL20 production and promote CRC in an orthotopic mouse model [107]. There are also other cell interactions with Treg cells, for example, Treg cells and mast cells are abundant in both human CRC and murine APC^∆468^ tumors, and the interaction between Treg-mast cells generates a suppressive-inflammatory Treg cell population that produces IL-17, which favors the expansion and degranulation of mast cells [108]. Taken together, these interactions between myeloid cells and Treg cells indicate an unfavorable prognostic.

### 6.7. Natural Killer (NK) Cells

On the contrary, NK cells have a cytotoxic role during myeloid leukemia, but their role in solid tumors is controversial because NK cells have limited infiltration [109]. It has been shown that CRC patients have an increased percentage of circulating Treg cells and reduced expression of NKp44 and NKp46 on both NK and NKT cells [110]. In chimeric mice lacking T cells and developing spontaneous intestinal tumors, it was demonstrated that the adoptive transfer of Treg cells and the NK cell depletion increased dramatically both the number and size of tumors with a decreased survival rate; this correlates with an impaired systemic production of IFN-γ (Figure 3B) [111]. Thus, there probably exists an interference of Treg cells over NK cells during CRC. This was demonstrated recently in a phase-1 clinical trial of adoptive transfer of expanded NK cells in combination with IgG1 antibody in patients with CRC and gastric cancer, in which this combinatory immunotherapy enhanced IFN-γ production and reduced peripheral Treg cells, and some patients showed an overall decrease in tumor size [112].

### 6.8. Invariant NK T Cells (iNKT Cells)

iNKT cells have an anti-tumor function and participate in the control of tumor metastasis through the secretion of IFN-γ [113]. Controversially, the absence of iNKT cells decreased the number of intestinal polyps in APC^min/+^ mice, correlating with a reduced frequency of Treg cells. Additionally, Ifn-γ and Nos2 genes were increased in polyps with increased frequencies of CD4^+^ and CD8^+^ T cells, suggesting that iNKT cells promote polyp formation in the intestine [114]. Different roles between either NK or iNKT cells have been demonstrated during CRC, and both impacted Treg cell activities.

## 7. Are Treg Cells a Good Target for Immunotherapy during CRC?

Depletion of Treg cells has been a target in clinical trials for some types of cancer with contrasting results. In melanoma, the administration of an IL-2/diphtheria toxin fusion protein to eliminate Treg cells apparently did not eliminate these cells or cause regression of metastatic melanoma [115]. However, the same protein fusion IL-2/diphtheria toxin significantly reduced the number of Treg cells in peripheral blood of patients with metastatic renal carcinoma and abrogated Treg-mediated immunosuppressive activity in vivo [116]. Some basic and clinical trials render strong evidence to show that Treg cells are therapeutic targets during CRC development. For example, low doses of an adenovirus expressing the IL-12 gene mediated a potent anti-tumor effect against subcutaneous colorectal carcinomas in mice in an immunosuppressive environment; this is caused by a direct effect on Treg cells, inhibiting in vitro secretion of IL-10 and TGF-β. Moreover, the treatment with the adenovirus-expressing IL-12 gene decreased the number of myeloid-derived suppressor cells (MDSC) and generated specific CD4^+^IFN-γ^+^ cells that were involved in the eradication of tumors (Figure 3B) [117]. Our own research suggests that Tim-3 is over-expressed in Treg cells in the CAC model [63]. It has also been suggested that Tim3^+^Foxp3^+^ Treg cells represent specialized tumor resident Foxp3^+^ cells that probably have a role in T cell dysfunction [35,118,119]. Supporting this last idea, it was demonstrated that Tim-3 is expressed on CD8^+^ tumor-infiltrating lymphocytes isolated from patients with CRC; these CD8^+^ T cells also co-expressed PD-1 and exhibited an exhausted phenotype because they did not secrete cytokines. Combined targeting of both Tim-3 and PD-1 with monoclonal antibodies increased the frequencies of IFN-γ and TNF-α and the proliferation of antigen-specific CD8^+^ T cells (Figure 3A). Additionally, with the use of these monoclonal antibodies, a decrease in Treg cells was observed [120]; thus, using either monoclonal antibodies or inhibitor molecules against Tim-3 could induce a protective response against CRC. The LAG-3 receptor is overexpressed in Treg cells from patients with CRC and liver metastasis, and an antibody blockade of LAG-3 increased the proliferation and effector cytokine production of intratumor T cells [121]. Another immunologic target is the CTLA-4, a molecule involved in the negative regulation of activated T cells; in Treg cells, its loss or inhibition results in reduced Treg cell function [122]. In a murine model, the use of anti-CTLA-4 antibody with IgG2a isotype exhibits enhanced antitumor activity in the colon adenocarcinoma tumor model; this effect was associated with a significant reduction in Treg cells at the tumor site, and, consequently, an expansion of activated CD8^+^ T cells was observed [123]. It has been demonstrated that low doses of cyclophosphamide target Treg cells in humans and animal models. In a clinical trial, 55 patients with metastatic CRC received 2 week-long courses of low-dose cyclophosphamide. An increased number of absolute T-cell numbers was found with a reduction in the percentage and absolute number of Treg, B, and NK cells. In addition, an increased amount of IFN-γ^+^ tumor-specific T cells and granzyme B were displayed, which was associated with a significant delay in tumor progression (Figure 3B) [124]. Together, these data strongly suggest that Treg cells are an immunotherapeutic target for CRC. We must also consider that immune-targeting Treg cells during CRC may affect some important oncogenes influencing the microenvironment of colorectal tissue such as p53, APC, or Kirsten Rat Sarcoma Viral (KRAS). Some recent studies have shown, for example, that patients with CRC microsatellite stable (MSS) disease do not respond effectively to PD-1 Immune Checkpoint Blockage (ICB), and this is caused by mutations on the oncogene KRAS, which induces the recruitment of MDSC and Treg cells [125]. Tumor cells carrying mutations in KRAS induce highly suppressive Treg cells and over-expression of KRAS^G12V^ gene-induced Treg cells [126]. APC deficiency in the mouse model reduces the presence of Nuclear Factor of Activated cells (NFAT) specifically in Treg cells, reducing differentiation and suppressive capacity [127]. Furthermore, it was suggested that p53 mutations in patients with CRC tumor recurrence is caused by a correlation between p53 and IL-10 from Treg cells [128]. All these observations make it difficult to believe that Treg cells by themselves are a unique suitable therapeutic target. Some studies in murine models and patients attempted to directly or indirectly narrow Treg cells to try to reduce the impact of tumorigenesis in either CRC or gastric cancer development, but these studies have failed because they suggested that CRC tumors are immuno-silent and hypo-responsive to ICB treatment [129,130,131]. For example, it was demonstrated that during gastric cancer, PD-1 blockade promoted an increased proliferation and suppressive activity of Treg cells [130]. To reduce this negative impact and to improve the efficacy of treatments in solid tumors, we and others have proposed the use of combined immunotherapy. For example, the use of Oxaliplatin (Folfox) in combination with anti-PD-1 antibodies in a murine model of CRC and in samples of patients reduced the number of tumors, which does not happen if these compounds are used individually [132]. The reduction in the number of tumors is caused by PD-1^+^CD8^+^T-bet^+^ cells infiltrating the tumor. Likewise, the protective immune response in the combined therapy of ICBs with Folfox was confirmed in another murine model of CRC, where the protection was CD8^+^ T-cell-dependent [133]. In the mouse CAC model, it was recently shown that the lack of STAT-6 transcription factor reduced the inflammation and generated protection; however, when STAT-6 activity was inhibited with a chemical drug (AS1517499), a partial, but significant, reduction in tumor development was observed [134]. Consequently, it is clear that a combined therapy using more than one target probably offers improved protection against the tumor generation during CRC, or at least has an increased likelihood of stopping CRC development. In fact, in a recent work targeting STAT-6 together with the inflammatory response and at the same time receiving the classical treatment of the drug 5-Fluorouracil, the authors were able to reverse previously established colon tumors using the CAC model [135]. These findings support the idea that targeting multiple molecules may be the best way to defeat this type of cancer.

## 8. Conclusions

There is strong evidence supporting the fact that the immune response plays a major role in CRC development, and some immunological interacting mechanisms create a complex network that leads to the development of polyps, adenomas, and tumors. Some years ago, we learnt lessons about complex immunological networks triggered during infectious diseases. We also learnt how to manipulate the host immune system for a better response during vaccine treatment. Now, we are applying these lessons to the development of cancer and, surprisingly, the facts that were described as having a role in the correct adaptation of parasites to the host are very similar to the causes of the development of tumors that lead to cancer. During CRC, tumors promote immune cell populations that favor immunosuppression; thus, tumors can be established without selective pressure. It is necessary to learn how we can manipulate this immunosuppressive microenvironment to choose the correct and timely action to modulate the immune response, which could probably (and hopefully) eradicate the tumor. We also need to learn how the reversion of immunosuppressive microenvironments during cancer would not cause autoimmunity or dangerous inflammatory responses. Here, the participation of Tregs appears to be crucial, but it is still controversial, given that removing Tregs may likely produce one of two disparate results: on the one hand, it could improve the anti-tumor immune response and accelerate tumor rejection; on the other hand, it could promote tumor establishment by releasing a long-lasting inflammatory response. The series of contrasting reports regarding Tregs in colon cancer described here tell us that we do not have enough information to determine which molecules associated with Tregs should be removed during CRC development, and whether they should be removed at the beginning or at advanced stages of CRC. Perhaps a combination of therapies that focus on different targets in either the same or another subpopulation of immune cells could be a first step to begin to win the battle against CRC.
